# Supplementary material for: Exploring healthcare-seeking behavior of most vulnerable groups amid the COVID-19 pandemic in the humanitarian context in Cox’s Bazar, Bangladesh: Findings from an exploratory qualitative study
Source: PLOS Glob Public Health. 2023 Mar 20;3(3):e0000382. doi: 10.1371/journal.pgph.0000382 (PMC10027208; doi:10.1371/journal.pgph.0000382)
Supplement: Attachment — Submitted filename: Response to Reviewers.docx [file pgph.0000382.s001.docx]

The Editor

PLOS Global Public Health

**Subject:** Rebuttal Letter for the Manuscript titled “Exploring healthcare-seeking behavior of most vulnerable groups amid the covid-19 pandemic in the humanitarian context in Cox’s Bazar, Bangladesh: findings from an exploratory qualitative study”

Dear Editor,

Thank you for your feedback that has helped us strengthen the manuscript. Here are the redressal and specific comments to your feedback:

|  | **Comment** | **Redressal** |
| --- | --- | --- |
| A. | Section: Journals requirements | |
| 1. | Please state in the Methods: Whether the Institutional Review Board (IRB) approved the use of oral consent | Thank you for the comment.  The comment has been addressed in lines number 162-163. |
| 2. | How oral consent was documented | Thank you for the comment.  The comment has been addressed in line number 169-170. |
| 3. | Please provide the full name of the Ethics Committee or IRB in the Ethics Statement section of the manuscript file | Thank you for the comment.  The comment has been addressed in lines number 162-163. Additionally, a citation to the IRB website of BRAC James P Grant School of Public Health has also been added. |
| 4. | Please send a completed 'Competing Interests' statement, including any COIs declared by your co-authors. If you have no competing interests to declare, please state "The authors have declared that no competing interests exist". | Thank you for the comment.  The comment has been addressed in lines number 24-25. |
| 5. | Please provide separate figure files in .tif or .eps format only and remove any figures embedded in your manuscript file. Please ensure that all files are under our size limit of 20MB. | Thank you for the comment.  The figures have been separately provided to meet the journal’s standard as mentioned. |
| 6. | For more information about how to convert your figure files please see our guidelines: Once you've converted your files to .tif or .eps, please also make sure that your figures meet our format requirements | The figure were converted according to the journal format |
| 7. | Please ensure that the funders and grant numbers match between the Financial Disclosure field and the Funding Information tab in your submission form. | Thank you for the comment.  The funders and grant numbers now match between the Financial Disclosure field and the Funding Information tab in our submission form. |
| B. | Section: Editors Comments | |
| 1. | Additional Editor Comments (if provided): Thank you for presenting a comprehensive descriptive and analytical account of the specific challenges faced by the Rohingya populations living in Cox's Bazar during the COVID-19 pandemic. It is a compelling and important issue; the rhetoric of equity and redistribution of meagre resources being under question, alongside the specific strategies used by MVGs when such pressures are exerted. I have one major issue with the manuscript as it stands. It is by far too long to be accessible for most readers. At over 10,000 words in length is more aligned to a full dissertation than a manuscript. I am therefore requesting the authors edit the manuscript length to reduce it to around 4,500 words (although I note that there is no word limit on submissions). | Thank you for the review and comments.  We have tried to cut the length of the manuscript to 6429 words (Excluding figures, and references) |
| 2. | Changes in Introduction | Thank you for the review. The introduction section has been reduced to 391 words. |
| 3. | The tables could be reduced to one or two (one in the methods, perhaps) and one to describe the sample (in the results). | Two tables from the methods section and result section have been removed per the suggestion of the reviewer. |
| 4. | The results are interesting and important, but again, some editing and merging of sections would only serve to improve the readability of the text. | Thank you for the review. The suggested changes have been made in the manuscript. |
| 5. | A clearer series of statements to describe the value this work adds to the broader literature, service response (background) and what this work will mean in the future (discussion) would again be of value to the reader. | Thank you for the review and comments  The value this work adds to the broader literature was described in lines number 98-102. |
| 6. | Abstract: How this work contributes to broader (or even national, regional) challenges and objectives for fragile and vulnerable populations during COVID-19 would anchor this substantial piece of work. | Thank you for the review and comments  contributes to broader (or even national, regional) challenges and objectives for fragile and vulnerable populations during COVID-19 described in lines number 69-73 |
| 7. | The Figure included in the background, does this need to be presented in the paper? Could it be referred to in the text and referenced? | Thank you for the comment. The authors discussed the figure and were in the consensus of keeping it in the manuscript as it presents the conceptual framework of our study and without one other table were removed. |

Thank you for providing us with a learning opportunity with these comments. We are hopeful that the pre-peer-review feedback has helped us reinforce our manuscript and concise it to comply with Journal’s aims and scope.

We look forward to hearing from you soon.

Thank you,

Best regards,

Corresponding Author

Riaz Hossain
