# Supplementary material for: Exploring healthcare-seeking behavior of most vulnerable groups amid the COVID-19 pandemic in the humanitarian context in Cox’s Bazar, Bangladesh: Findings from an exploratory qualitative study
Source: PLOS Glob Public Health. 2023 Mar 20;3(3):e0000382. doi: 10.1371/journal.pgph.0000382 (PMC10027208; doi:10.1371/journal.pgph.0000382)
Supplement: Attachment — Submitted filename: Response to Reviewers.docx [file pgph.0000382.s002.docx]

The Editor

PLOS Global Public Health

**Subject:** Rebuttal Letter for the Manuscript titled “Exploring healthcare-seeking behavior of most vulnerable groups amid the covid-19 pandemic in the humanitarian context in Cox’s Bazar, Bangladesh: findings from an exploratory qualitative study”

Dear Editor,

Thank you for your feedback, which has helped us strengthen the manuscript. Here are the redressal and specific comments to the reviewer’s feedback:

|  | **Comment** | **Redressal** |
| --- | --- | --- |
| A | **Comments from reviewer 1** |  |
| 1. | Introduction  Since the situation of Rohingya is not very familiar to all readers, I would suggest that a little more background for the study should be written in the introduction section (or settings). For example, where do Rohingyas come from, when did they arrive in Bangladesh and why. A little more information about the population group, such as religion, is needed. The same applies to the local host population, as there is little information about its background. It would also be good to describe the relations between these two communities. Adding such information helps the reader better understand the research results and put them in context. | Thank you for the comment.  The comment has been addressed in line numbers 73 to 78 |
| 2. | line 85: define the abbreviation WASH | Thank you for the comment.  The comment has been addressed in line number 89 |
| 3. | Materials and Methods  line 122: authors say that they have conducted an exhaustive literature review. However, the article does not present other studies that are related to the topic. It would be desirable to highlight a few other such studies. The same issues have been researched in other conflict settings, such as in Syria. (Agneta KALLSTRÖM \| University of Eastern Finland) give some citations from her publication | Thank you for the comment  The comment has been addressed in line number 579-580, and 602 |
| 4. | line 127: Even though Andersen's model is well known, the authors could briefly describe the method. | Thank you for the comment.  A brief description was provided in line number 135-138. |
| 5. | - lines 129-130. The authors stated that they had left certain factors out of the analysis, such as religion (in case of refugees it is Islam). | Thank you for the comment.  The factor was incorporated into the model. |
| 6. | line 129: Figure 1. is missing ….our exploration of factors influencing healthcare-seeking behavior in the Rohingya and host community contexts (Fig 1) | Figure 1 was given in a separate JPG file following the journal guideline. |
| 7. | The study lacks information about how the interviewees were selected for the study. At what point was it decided to stop continuing the interviews? Is the sampling reliably representative of the whole population and likely to be without biases? I think this an important consideration that should be added. | Thank you for the comment.  The comment has been addressed in line number 148-152. |
| 8. | Results  lines 222 - 224 As a result, many of them admitted seeking help 223 from their male family members whenever they faced any health-related issues or menstrual kit. Here should be “ or when women needed the mensural kit? | Thank you for the comment.  The comment has been addressed in line number 234-235 |
| 9. | lines 264- 272 I think this is background info and should be transferred to context / setting chapter | Thank you for the comment.But line 276 to 277 are needed here to set the background to present the result from line number 279 to 286. |
| 10. | line 344 paled: shold be placed? | Thank you for the comment.  The comment has been addressed in line number 356 |
| 11. | - lines 456 - 457 Most of their treatment was from the paracetamol group, and doctors prescribed those without proper examination. Due to this reason, patients were not recovering as early as the pre-pandemic period. This sentence is a bit problematic. Providing paracetamol has been almost the only possible medication for symptoms of COVID-19 since there is no cure for the disease. This argument shows that the patients did not know the facts about the virus. The interviewees felt that they did not receive proper treatment (because they did not know that it did not exist). While failing to provide life-sustaining medical aid to critically ill patients would constitute a medical performance failure, giving paracetamol as the typical treatment for patients without complications is actually proper practice. Objectively sub-par treatment should be separated from the subjective experience of the patients. In itself this phenomenon could be an interesting point for further study. Patients might have appreciated the treatment better had they been informed about COVID transmission and feasible medication. | Thank you for pointing out this subjective issue.  The comment has been addressed in line number 466-470 |
| 12. | - It was noted at several points that financial issues were one factor limiting the availability of medical services. It would be helpful to the reader if the funding structure of different healthcare services was described in more detail. For which services the patients themselves were expected to pay? Was lack of state or NGOs funds a significant factor in this sense? | Thank you for the comment.  This research project did not collect information on the financial structure of various healthcare services, which differs depending on the entity that runs the health facilities.  In the camps' healthcare center constructed by international organizations, Rohingyas received free medical care and medicines. Also, in the host community, government health facilities charged only the bare minimum, and most recommended medications were unavailable. For better treatment, medicine, and medical test, participants have to visit private clinics. |
| 13. | Discussion and Conclusion The findings appear to be logical and believable, consistently supporting previous findings. There are some parts of the discussion where telling apart findings of previous publications from the ones done in this study is challenging. It would be beneficial to be explicitly clear about the new core findings in this section. Also, the results could be connected to an even broader perspective by comparing them against comparable studies from contemporary conflict settings such as Syria. It was noted that the results could not be generalized to other Rohingya camps and host communities. What is the maximum extent of reliable generalizability of the results, and what are the limiting factors? Should the article be viewed primarily as a case study? | Thank you for the comment.  The comment has been addressed in line number 559-562  The comment on the generalizability has been addressed in line number 564-568 |
| B | **Comments from Reviewer 2** |  |
| 14. | In the abstract it says, “This study adopted the modified version of Anderson’s behavioral model”. Did the authors further modify the Anderson’s behavioral model? because the authors stated “… However, from our study, it was found that some factors such as religion, occupation, residence, etc., were deemed irrelevant in our context and were thus deleted from our conceptual framework”. Also what factors were included in the “etc.”? How the authors did this modification, and then decided to exclude some factors and include a new factor into the conceptual framework? | Thank you for the comment  The comment has been addressed in line number 42 and more details were given in line number 131-136. |
| 15. | Sampling and Data Collection Table1 can be part of the analysis (summarized as well). The topic sentence could be “Data were collected from men and women, aged 11-65+ years, from the Rohingya and host communities, using in-depth interviews conducted between Nov 2020 and Mar 2021. The following sentences can be moved to analysis “In both communities, 48 in-depth interviews ……, and sampling.” | Thank you for the comment  The comment has been addressed in line number 144-147 |
| 16. | Under the subsection of “perceived benefit”, at the end of paragraph it is written “In all cases, most female adult participants expressed a strong understanding of the severity of COVID19). Unless “perceived benefit” is defined, a reader may not understand whether this sentence is related to participants’ knowledge, or perceived severity of illness or perceived benefit. | Thank you for the comment  The information was removed. |
| 17. | A reference is needed for “…. decision to redirect resources to COVID-19 reaction [ref]” | Thank you for the comment  The information was removed. |
| 18. | The authors may opt to see the Health Belief Model | Thank you for your remark. However, we conclude that the Andersen behavior model best fits the analysis. |
| 19. | The paragraph “The findings revealed that in both study communities …” under the subsection of “Age, Sex, and Family Income” is confusing. | Thank you for the comment  The information was updated in line number 205 |
| 20. | Therefore, religion is a key factor, and should be included in the conceptual framework. | Thank you for the comment  Added in the framework under health belief factor. |
| 21. | Discussion The authors may opt to organize the discussion a bit clearly, as currently this isn’t the case. The first paragraph seems redundant. The authors can start with “In this qualitative study of 48 men and women, aged 11-65+ years, from the Rohingya and local communities of the Cox’s Bazar we found that mobility restriction was a barrier for …”. The first paragraph should be an overall summary of the findings. | Thank you for the comment  The comment has been addressed in line number 499-502 |
| 22. | There are repetitions and redundant sentences which the authors need to address them (for example, “As seen in our data” is a redundant phrase. | Thank you for the comment  The comment has been addressed in line number 381, 390 |
| 23. | (According to the study findings, factors like perceived benefit and source of health information or disinformation were found to be some of the key individual factors that determined the healthcare seeking behaviour of our participants) can be shortened | Thank you for the comment  Rephrased in line number 347-349 |
| 24. | Also the word “key” is confusing; thus, the authors should report what they found, and if they use “key individual factors” then the question will be “what were the factors that were not key factors?” | Thank you for the comment  Rephrased in line number 347-349 |
| 25. | Please use the full description for any abbreviation used the first time in a sentence; for example, “WASH” was used for the first time in the introduction without describing it in full. | Thank you for the comment.  The comment has been addressed in line number 89 |
| 26. | Please use the past tense instead of present tense for what you did in this study; for example, instead of “… we elicit evidence…” we elicited evidence, OR instead of “This study utilizes a subset …” This study utilized a subset … Please revise “… Data was collected …” to “… Data were collected …” | Thank you for the comment  The comment has been addressed in line number 126, 130, 146 |
|  | Please consider “Distance to health facilities” instead of “Distance from health facilities” | Thank you for the comment  The comment has been addressed in line number 383, 415-16 |

Thank you for providing us with a learning opportunity with these comments. We are hopeful that the pre-peer-review feedback has helped us reinforce our manuscript and concise it to comply with Journal’s aims and scope.

We look forward to hearing from you soon.

Thank you,

Best regards,

Corresponding Author

Riaz Hossain
