# Supplementary material for: Exploring healthcare-seeking behavior of most vulnerable groups amid the COVID-19 pandemic in the humanitarian context in Cox’s Bazar, Bangladesh: Findings from an exploratory qualitative study
Source: PLOS Glob Public Health. 2023 Mar 20;3(3):e0000382. doi: 10.1371/journal.pgph.0000382 (PMC10027208; doi:10.1371/journal.pgph.0000382)
Supplement: Attachment — Submitted filename: Response to Reviewers.docx [file pgph.0000382.s003.docx]

The Editor

PLOS Global Public Health

**Subject:** Rebuttal Letter for the Manuscript titled “Exploring healthcare-seeking behavior of most vulnerable groups amid the covid-19 pandemic in the humanitarian context in Cox’s Bazar, Bangladesh: findings from an exploratory qualitative study”

Dear Editor,

Thank you for your feedback, which has helped us strengthen the manuscript. Here are the redressal and specific comments to the reviewer’s feedback:

|  | **Comment** | **Redressal** |
| --- | --- | --- |
| A | **Comments from reviewer 3** |  |
| 1. | The authors have addressed previous reviewer comments satisfactorily.  There is however an issue with the data availability statement - the authors state that all data is presented in the manuscript, which seems very unlikely given they interviewed 48 people. The authors should use the guidelines for qualitative data here: https://journals.plos.org/globalpublichealth/s/data-availability to complete their data availability statement. As it's common that qualitative data is subject to sharing restrictions given the identifiable and often sensitive nature of the data, the authors can use this statement to explain any restrictions on data sharing (identifiability, data sharing not covered by participants' consent). | The available data includes illustrative quotes drawn from the transcripts, and these are presented within the paper. All qualitative full transcripts are unavailable to the public because they contain personal identifying information about Rohingya and host participants and very sensitive information concerning Rohingya individuals seeking health care outside the camps, which is restricted. Ethical approval for the conduct of the study required that all data, including the locations of Rohingya participant camps, be de-identified, imposed by Institutional Review Board (IRB) - Research - brac jpgsph, <mailto:irb-jpgsph@bracu.ac.bd>. For data access, qualified researchers may use the following point of contact: Bachera Aktar, Assistant Director, The Center of Excellence for Gender, Sexual and Reproductive Health and Rights (CGSRHR), <bachera.aktar@bracu.ac.bd>, BRAC James P Grant School of Public Health, BRAC University 6th Floor, Medona Tower, 28 Mohakhali Commercial Area, Bir Uttom A K Khandakar Road, Dhaka-1213, Bangladesh. |

Thank you for providing us with a learning opportunity with these comments. We are hopeful that the pre-peer-review feedback has helped us reinforce our manuscript and concise it to comply with Journal’s aims and scope.

We look forward to hearing from you soon.

Thank you,

Best regards,

Corresponding Author

Riaz Hossain
